# Usage and Preferences of Orthodontic Mini-Implants Among Romanian Practitioners: A Survey Study

**DOI:** 10.3390/dj12120400

**Published:** 2024-12-06

**Authors:** Teodora Consuela Bungău, Abel Emanuel Moca, Gabriela Ciavoi, Ioana Mihaela Romanul, Luminița Ligia Vaida, Camelia Liana Buhaș

**Affiliations:** 1 Department of Dentistry, Faculty of Medicine and Pharmacy, University of Oradea, 410073 Oradea, Romania; consuela.bungau@uoradea.ro (T.C.B.); ioana_romanul@uoradea.ro (I.M.R.); ligia_vaida@uoradea.ro (L.L.V.); 2 Department of Morphological Disciplines, Faculty of Medicine and Pharmacy, University of Oradea, 410087 Oradea, Romania; cameliabuhas@uoradea.ro

**Keywords:** orthodontic mini-implants, skeletal anchorage, orthodontic treatment

## Abstract

**Background/Objectives:** Dental malocclusions are highly prevalent worldwide, negatively impacting patients’ quality of life and leading to complex, often costly, orthodontic treatments. In Romania, the economic status of patients and the limited public funding for orthodontic care significantly influence treatment accessibility and choices. Advanced technologies, such as mini-implants (MIs), offer improved anchorage and treatment efficiency but are often underutilized due to financial constraints and variability in clinical training. In this context, there are limited data regarding the use and preferences of MI among orthodontists in Romania. This study aims to explore the characteristics, preferences, and challenges of Romanian orthodontists in their use of MI systems. **Methods:** A survey was conducted between June and September 2024, targeting orthodontists across Romania. The questionnaire, distributed via social media platforms, consisted of 24 items addressing professional experience, MI system preferences, insertion methods, and complications. Statistical analyses were conducted using IBM SPSS Statistics 25. Fisher’s Exact Test and Pearson’s Chi-Square Test were employed to evaluate relationships between categorical variables. When appropriate, logistic binomial univariable regression models were applied to predict key dependent variables (e.g., MI placement zones, MI experience, and MI complications) based on independent variables such as specific MI system usage and frequency of MI usage. A significance threshold of α = 0.05 was used for all tests. **Results:** Out of 105 participants, 85.7% reported using mini-implants (MIs) in their orthodontic practice, with the Dual Top and Benefit systems being the most frequently used (60% and 43.3%, respectively). The interradicular area was the most common placement site (60%), while the palatal and retromolar regions showed significant correlations with the Benefit system (*p* = 0.008). Practitioners with more than 10 years of experience reported a significantly higher frequency of MI use (*p* = 0.001), with frequent use being observed in 60.9% of these practitioners. Complications were common, with MI mobility reported by 92.2% and soft tissue damage by 57.8%. The midpalatal area was significantly associated with higher complication rates compared to other sites (*p* < 0.001). The success rates of MI usage ranged from 76% to 100% in 57.8% of respondents, with higher success rates being associated with infrazygomatic placements (*p* < 0.05). **Conclusions:** MI usage is prevalent among Romanian orthodontists, with experienced practitioners utilizing them more frequently. Despite high success rates, common complications highlight the need for improved insertion techniques and post-operative care. Further research and training are recommended to optimize MI application and reduce complication rates.

## 1. Introduction

Dental malocclusions are highly prevalent worldwide, affecting approximately 56% of the population globally and up to 72% in Europe [1]. These conditions are complex and challenging to classify due to their varied etiology and manifestations [2]. Dental malocclusions negatively affect patients’ quality of life [3], and the orthodontic treatments required can vary significantly in complexity [4]. These treatments may also impose a substantial economic burden on patients or their families [5]. To ease treatment for patients, preserve the integrity and functionality of the dento-maxillary apparatus, and reduce treatment time—and, consequently, the costs of orthodontic care—new orthodontic treatment methods are being developed and implemented [6].

Orthodontic mini-implants (MIs), also known as Temporary Anchorage Devices (TADs), are part of this orthodontic advancement. They represent an effective method for achieving skeletal anchorage, offering numerous advantages such as ease of placement and removal, a small size, increased patient comfort, the absence of surgical defects, and immediate loading [7,8]. MIs have a wide range of applications, including their use in treatments requiring intrusion, extrusion, retraction, distalization, or mesialization [8,9], as well as in the management of rare and severe dento-skeletal disorders [9]. Given their numerous advantages, MIs have become an increasingly popular therapeutic option among orthodontists [10]. The growing market demand has been met by manufacturers, and a wider variety of MIs are now available. These implants typically vary in length from 4 to 12 mm and are categorized as small, medium, or large. Additionally, their diameters vary, with the most commonly used MIs having diameters ranging from 1.15 to 2.5 mm. The designs of the head and screw also vary, taking on different forms [11].

The insertion protocol is generally based on a clinical examination of the intended insertion site combined with 2D or 3D imaging [12]. For even greater control during insertion, computer-aided design (CAD) can be used to manufacture custom guides [13]. Thorough planning is essential to increase the success rate of MIs, as failures can occur due to patient-related factors (e.g., gender, age, oral hygiene, and placement site), MI-related factors (e.g., insertion site, size, and design), or operator-related factors [14]. However, the literature on the effects of operator-related aspects, such as clinical experience, frequency of MI use, and specialist qualifications, is limited.

In Romania, data on the use of MI—particularly regarding the operators, systems used, instruments employed, evaluation methods, and most frequent application sites—are lacking at the time of this manuscript’s writing. Nevertheless, the prevalence of dento-maxillary anomalies is high and rising [15], suggesting that MI usage is likely to increase among Romanian orthodontists. Romanian orthodontic practices are influenced by distinct clinical, educational, and economic factors, which differ from those in Western Europe, Asia, and North America [16]. Patient expectations and financial constraints in Romania also shape treatment choices, diverging from practices in countries with greater healthcare funding or differing regulatory systems [17].

Therefore, to gain an overview of the characteristics and preferences of Romanian orthodontists who use MIs, the aim of this study was to identify the main characteristics of these professionals (e.g., professional experience, frequency of MI use, and specialist qualifications) as well as their preferences regarding MI systems, instruments, and evaluation methods used prior to insertion. Moreover, this study provides insights tailored to the Romanian context, highlighting practical recommendations and supporting evidence to refine and advance local practices in orthodontic mini-implant use.

## 2. Materials and Methods

### 2.1. Ethical Considerations

This research was conducted in accordance with the principles outlined in the 1964 Declaration of Helsinki and its subsequent amendments. This study was approved by the Ethics Committee of the Faculty of Medicine and Pharmacy, University of Oradea (No. CEFMF/04, dated 4 February 2022). Prior to completing the questionnaire, respondents were informed about the purpose of the survey as well as the fact that participation was voluntary and anonymous, with no financial or other incentives offered. Consent to participate in the study was implicitly given through the voluntary completion of the questionnaire.

### 2.2. Participants and Data Collection

This study was conducted as a survey over a three-month period from 1 June 2024 to 1 September 2024. The questionnaire, designed by the authors, was uploaded and distributed using the Survio platform (Survio s.r.o., Brno, Czech Republic). The questionnaire link was shared across various social media platforms within groups related to dental medicine and orthodontics in Romania to maximize reach.

The primary outcome of this study was the frequency and patterns of mini-implant (MI) usage among Romanian orthodontists, including their preferences for MI systems, insertion methods, and complications encountered. The primary independent variable of interest was practitioners’ professional experience, categorized by years in practice (<5 years, 5–10 years, 11–15 years, 16–20 years, and >20 years).

Covariates considered in the analysis included the following:

- Gender (male or female);
- Specialization (e.g., orthodontics, dento-alveolar surgery, etc.);
- Geographic location (city of practice);
- Specific MI systems used (e.g., Dual Top, Benefit, OrthoEasy, etc.);
- Frequency of MI usage (e.g., very frequently, frequently, occasionally, rarely, or very rarely);
- Preferred MI placement zones (e.g., interradicular, palatal, retromolar, etc.);
- Reported complications (e.g., MI mobility, soft tissue damage, MI fracture, etc.).

The questionnaire consisted of 24 items, which were divided into the following four main sections:

- Section One: This section included the first four items, aimed at gathering information about the respondent. It collected data on gender (male or female), specialization (orthodontics, dento-alveolar surgery, general dentistry, oral and maxillofacial surgery, other specializations, or resident doctor), years of professional experience (less than 5 years, 5–10 years, 11–15 years, 16–20 years, or over 20 years), and the city in Romania where the practitioner works. The first three items required the respondent to choose one of the available options, while for the fourth item, respondents were asked to freely indicate the city in which they practice.
- Section Two: This section, comprising items 5–12, investigated the practitioner’s experience with MIs. Respondents were given multiple-choice options to select their answers. The specific questions and their corresponding response options are presented in Table 1.

- Section Three: This section included items 13–19 and collected information on the dimensions of MIs used in various anatomical regions, such as the maxillary interradicular area (item 13), mandibular interradicular area (item 14), infrazygomatic area (item 15), midpalatal area (item 16), palatal area (item 17), maxillary retromolar area (item 18), and mandibular retromolar area (item 19). Respondents provided open-ended answers to these items.
- Section Four: This section comprised items 20–24, which gathered data on the operator’s satisfaction with MIs and included an assessment of the risk of complications. The items in this section, along with the available response options, are presented in Table 2.

This study included doctors practicing in Romania who are actively involved in orthodontic practice, either through the use of MIs or as specialist orthodontists and residents who perform orthodontic treatments. Given the focus of the research on MI usage, it was crucial to include practitioners with direct or potential experience in the field of orthodontics, allowing for a comprehensive assessment of MI application across varying levels of expertise. The criteria were designed to encompass a broad spectrum of orthodontic professionals, ensuring that the study captured a wide range of insights on the practical challenges and preferences related to MI.

Participants who did not meet the qualifications to practice orthodontics in Romania or those who worked outside of the country were excluded from the study as the research aimed to focus on the specific practices and preferences within Romania’s professional environment. Additionally, doctors from specialties other than orthodontics who do not use MI were also excluded to maintain the study’s focus on the intended population. This decision was made to ensure that the data collected would be directly relevant to the clinical use and evaluation of MIs in orthodontics. Moreover, practitioners who neither use mini-implants nor plan to incorporate them into their practice were excluded unless they were orthodontic residents or specialists in the field.

The sample size calculation was performed using Python 3 (Python Software, Python Software Foundation, Wilmington, DE, USA Foundation, Wilmington, DE, USA). To determine the appropriate sample size for the study, we aimed for a 95% confidence level, corresponding to a Z-score of approximately 1.96, and a margin of error of 5%, which is a common standard in scientific research to balance precision and feasibility. Assuming a population proportion of 0.5, which yields the maximum required sample size, the initial sample size was calculated using the following formula:

(1) $$n=\frac{Z^{2\;}\cdot p\;\cdot (1-p)}{E^{2}}$$

where *Z* is the Z-score, *p* is the population proportion, and *E* is the margin of error.

Since the total population size of the Romanian Association of Excellence in Orthodontics (AREO) was finite (*N* = 123), the sample size was adjusted using the finite population correction equation:

(2) $$n_{adjusted}=\frac{n}{1+\frac{n-1}{N}}$$

where *N* is the population size.

Using this method, the final adjusted sample size was calculated to be approximately 94 participants. This sample size ensures the results are representative of the target population with the specified confidence level and margin of error.

### 2.3. Statistical Analysis

Statistical analyses were conducted using IBM SPSS Statistics 25, with the results visualized in Microsoft Excel and Word 2021. Qualitative variables were presented as absolute values and percentages to summarize the data distribution. Differences between groups were assessed using Fisher’s Exact Test or Pearson’s Chi-Square Test depending on data characteristics and sample size. Logistic binomial univariable regression models were applied where appropriate to predict key dependent variables (e.g., MI placement zones, MI experience, and MI complications) based on independent variables (e.g., MI system type and frequency of MI usage). Models were validated through significance testing and goodness-of-fit assessments. Odds ratios with 95% confidence intervals were calculated to evaluate the performance of predictions and the significance of independent variables.

To explore significant associations identified in contingency tables, Z-tests with Bonferroni correction were used to adjust for multiple comparisons and minimize the risk of Type I errors, ensuring robust results. The statistical methods employed provided a comprehensive approach to analyzing the data and validating the study’s findings. A significance threshold of α = 0.05 was applied for all tests.

## 3. Results

### 3.1. Section One: Socio-Demographic Characteristics

A total of 105 dentists participated in the questionnaire concerning the use of MI in orthodontic treatments. The respondents were diverse in terms of gender, with a predominant representation of female professionals. Specifically, 70.5% (*n* = 74) of the participants were women, emphasizing a notable female presence in this field, while the remaining 29.5% (*n* = 31) were men.

In terms of specialization, the majority of the respondents were orthodontic specialists (88.6%, *n* = 93), with 32.4% (*n* = 34) having less than 5 years of professional experience. However, approximately one-quarter of the respondents had between 11 and 15 years of experience (25.7%, *n* = 27). Most participants were based in Oradea, accounting for 23.8% (*n* = 25) of the sample, followed by Cluj-Napoca with 18.1% (*n* = 19) and Bucharest with 15.2% (*n* = 16). The distribution of patients according to these variables is detailed in Table 3.

### 3.2. Section Two: Practitioners’ Experience with Mini-Implants (MIs)

Out of the 105 dentists who completed the questionnaire, 85.7% (*n* = 90) reported incorporating MI into their orthodontic treatment protocols, while only 14.3% (*n* = 15) indicated that they do not use MI. Among those who utilize MI, the majority have been using them for either 1–3 years (26.7%, *n* = 24) or for more than 10 years (25.6%, *n* = 23).

The most commonly used mini-implant systems were Dual Top (60%, *n* = 54), Benefit (43.3%, *n* = 39), OrthoEasy (14.4%, *n* = 13), and OrthAnchor (23.3%, *n* = 21). According to the results, in most cases, the orthodontist is primarily responsible for the placement of the MI (78.9%, *n* = 71), sometimes in collaboration with a dentoalveolar surgeon (40%, *n* = 36). Regarding the site of mini-implant placement, the interradicular area was the most common (60%, *n* = 54). Responses to other items in this section are detailed in Table 4.

Statistically significant results were observed regarding MI placement and system usage. The Dual Top system was strongly associated with a higher frequency of MI placement in the palatal area (24.1% vs. 2.8%; Fisher’s Exact Test: *p* = 0.004; LR: OR = 11.098; 95% CI: 1.382–89.129; *p* = 0.024). Similarly, a significant relationship was found between the Benefit (PSM) system and placement in both the palatal and retromolar regions (Fisher’s Exact Test: *p* = 0.008). Benefit (PSM) users showed a higher preference for these regions, with placement rates of 25.6% vs. 7.8% for the palatal region (LR: OR = 4.052; 95% CI: 1.163–14.120; *p* = 0.028) and 12.8% vs. 2% for the retromolar region (LR: OR = 7.353; 95% CI: 0.822–65.752; *p* = 0.074). While the latter showed a trend toward statistical significance, it suggested a higher preference for retromolar placement among Benefit (PSM) users.

Z-tests with Bonferroni correction further revealed significant associations between specific MI systems and placement sites. Retromolar applications (80% vs. 2.4%) were strongly associated with the Fatscrew system (Air Orthodontics) (Fisher’s Exact Test: *p* < 0.001; LR: OR = 166; 95% CI: 12.308–2238.78; *p* < 0.001). The infrazygomatic area (50% vs. 8.1%) was significantly linked to the Vector TAS system (Ormco) (Fisher’s Exact Test: *p* = 0.028; LR: OR = 11.286; 95% CI: 1.373–92.796; *p* = 0.024), as shown in Table 5.

The association between the frequency of MI usage and the duration of their use in practice was analyzed (Table 6). A statistically significant relationship was observed (Fisher’s Exact Test: *p* = 0.001). Frequent use of MI was the most common among participants with over 10 years of experience (39.1%) and, to a lesser extent, among those with 1–3 years of experience (16.7%). Over 40% of participants with 3–10 years of experience reported frequent use, which increased to 60.9% among those with over 10 years of experience. Occasional use was primarily reported by those with less than 1 year (50%) or 1–3 years (37.5%) of experience. Rare and very rare use was mostly limited to participants with fewer than 10 years of experience. Notably, no participants with more than 10 years of experience reported occasional, rare, or very rare use of MIs.

**Figure 1:**
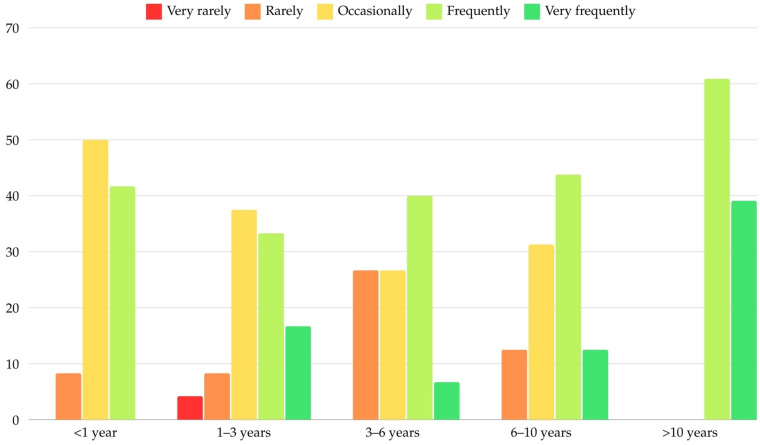
Distribution according to frequency and MI usage experience.

When MI usage frequency was recoded into two categories (frequently/very frequently vs. occasionally/rarely/very rarely) and the duration of MI usage was categorized as less than or greater than 3 years, a significant association persisted. Participants who used MIs frequently or very frequently were significantly more likely to have been using MIs for over 3 years (72.2%, 39 cases vs. 47.2%, 17 cases; *p* = 0.026; LR: OR = 2.906, 95% CI: 1.200–7.039, *p* = 0.018). Figure 1 summarizes the frequency of MI usage across different levels of practitioner experience with MI.

### 3.3. Section Four: Practitioners’ Experience with MI and Complications of MI

Section four consisted of questions 20 to 24. The majority of practitioners reported that the success rate of MI in their practice was between 76% and 100%, as indicated by 57.8% of respondents (*n* = 52). Additionally, there was a low overall risk of complications associated with MI use. According to the results, the most frequently cited reasons for using MI were improved anchorage stability (77.8%, *n* = 70) and the ability to manage complex cases (75.6%, *n* = 68).

However, complications related to MI use were also reported. The most commonly observed issues included MI mobility (92.2%, *n* = 83), soft tissue damage (57.8%, *n* = 52), and pain or discomfort following insertion (54.4%, *n* = 49) (Table 7).

**Figure 2:**
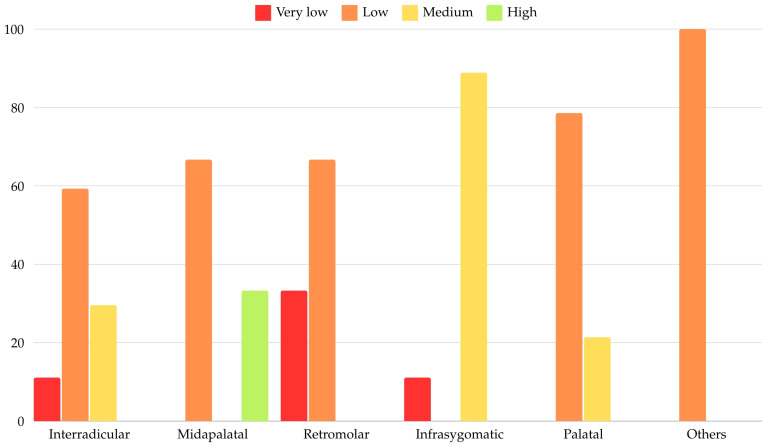
Distribution according to MI placement and overall risk of complications.

Regarding the statistical significance of the findings in this section, an association was identified between the overall risk of complications and the insertion site of the MI, according to Table 8. The differences observed between groups were statistically significant according to Fisher’s Exact Test (*p* < 0.001). Z-tests with Bonferroni correction revealed the following: cases with a higher overall risk of complications were significantly more frequently associated with MI placement in the midpalatal area compared to the interradicular area (33.3% vs. 0%). Logistic regression models could not be computed for this association due to the small number of cases for most subgroups. Figure 2 illustrates the distribution of MI placements in relation to the overall risk of complications.

As shown in Table 9, MI placement in the retromolar region was significantly associated with a higher occurrence of fractures (26.3% vs. 1.4%; *p* = 0.016; LR: OR = 25; 95% CI: 2.709–230.734; *p* = 0.005). In contrast, MI placement in the interradicular region was significantly less frequently associated with hard tissue damage (63.7% vs. 30%; *p* < 0.001; LR: OR = 0.244; 95% CI: 0.058–1.016; *p* = 0.053). Although the logistic regression model indicated a trend toward statistical significance, it suggested lower odds of hard tissue damage in cases of interradicular MI placement.

**Figure 3:**
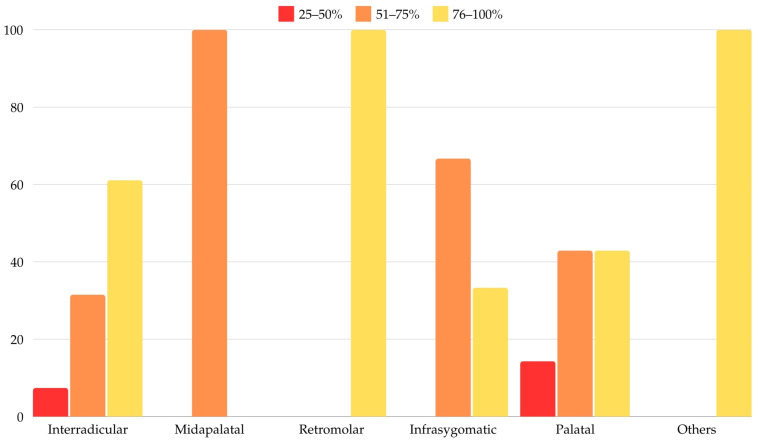
Distribution according to MI placement and overall success rate.

Additionally, success rates between 76% and 100% were significantly more frequently associated with MI placement in the retromolar region compared to the midpalatal area (100% vs. 0%; *p* = 0.038), as presented in Table 10. Logistic regression models could not be computed for this association due to the small number of cases for most subgroups. Figure 3 shows the distribution of MI placements in relation to overall success rates.

## 4. Discussion

The findings of this study provide an overview of the use of orthodontic MI among Romanian orthodontists, offering valuable insights into practitioners’ preferences, experiences, and challenges associated with MI usage. With 85.7% of respondents reporting the use of MI in their practice, it is evident that these devices play a significant role in contemporary orthodontic treatment in Romania. The adoption rate of MIs in Romania is significantly higher compared to that reported in other countries. For instance, a 2020 survey found that 65.8% of Canadian orthodontists incorporate TADs/MIs in their clinical practice [18]. In India, only 43.7% of orthodontists reported using MIs according to a study by Meeran et al. in 2012 [19]. Similarly, a survey conducted in 2015 by Bock and Ruf revealed that just 50% of German orthodontists regularly utilized skeletal anchorage devices in their practice [20]. In Saudi Arabia, 74% of orthodontists reported using MIs [21], whereas a survey in the United States indicated that nearly 70% of USA orthodontists employed MIs in private practice [22]. It is worth noting, however, that some of the comparative data from other countries are based on older studies. Given the rapid advancements in orthodontic technologies, it is plausible that the usage rates of MIs in these countries have increased since the original surveys were conducted.

In terms of clinical experience with MIs, 26.7% of practitioners in Romania reported using them for 1–3 years, while 25.6% indicated having over 10 years of experience. This distribution suggests that both early adopters and newer practitioners are actively integrating MIs into their clinical workflows. Similar trends are observed in other countries. For instance, in Canada, most orthodontists reported using MIs for 6–10 years [18], whereas in Saudi Arabia [21] and India [19], the majority had less than 3–5 years of experience. The diverse range of experience levels among Romanian orthodontists underscores a widespread and comprehensive adoption of MIs across multiple generations of practitioners. This pattern suggests a collaborative trend, where both seasoned professionals and emerging clinicians recognize and leverage the benefits of MIs. Consequently, this blend of expertise may contribute to Romania’s notably higher adoption rate compared to other countries.

The most commonly used MI system among Romanian practitioners is the Dual Top system, followed by the Benefit system. The high usage of these systems may be related to their favorable design features and the increased stability reported by practitioners. While a few systems dominate the Romanian market, there is also a diversity of choices reflecting different preferences and needs among orthodontists. Additional studies would be necessary to assess the exact market share of orthodontic mini-implant systems across Europe for comparative reasons.

Interestingly, this study found a significant correlation between the MI system used and the placement site. The Dual Top system was associated with increased usage in the palatal and interradicular area, while the Benefit system was more frequently used in both palatal and retromolar regions. Additionally, Fatscrew system users also demonstrated a higher preference for retromolar areas. These findings may reflect the design characteristics of each system, which could be optimized for different anatomical regions. Such preferences underscore the importance of system-specific training and experience in maximizing the effectiveness of MI placement, particularly in challenging areas like the interradicular, palatal, or retromolar region [23,24,25].

One of the key findings of this study is the relationship between MI usage frequency and the operator’s clinical experience. Practitioners with over 10 years of experience were more likely to use MI frequently, while those with less than 3 years of experience reported occasional or rare use. This trend suggests that more experienced practitioners may be more confident in utilizing MIs for a wider range of clinical scenarios, likely due to their familiarity with the devices and their associated protocols. In contrast, less experienced practitioners may still be building their confidence or may prefer traditional anchorage methods in less complex cases.

Romanian orthodontists predominantly place MIs themselves, with 78.9% reporting personal involvement in the procedure. This high rate of self-placement is particularly noteworthy given that many Romanian orthodontists do not work in large interdisciplinary clinics where dentoalveolar surgeons are readily available. In the absence of an on-site surgical specialist, orthodontists often need to acquire the necessary skills to independently place MIs, ensuring timely and efficient patient care. By personally performing MI placement, Romanian orthodontists can retain greater control over the treatment plan and its execution, potentially leading to improved clinical outcomes. Similar trends are observed internationally. In Canada, 72% of orthodontists reported personally placing MIs [18]. In Saudi Arabia, this figure is even higher, with 80% of orthodontists performing the procedure themselves [21]. However, lower self-placement rates are observed in other regions, such as Germany, where legal regulations and varying comfort levels with surgical procedures influence practice patterns. According to Bock and Ruf (2015), only 2% of German orthodontists reported placing MIs themselves, with the majority delegating the procedure to oral surgeons or maxillofacial specialists [20]. These differences may reflect variations in training protocols, regulatory frameworks, and practitioners’ confidence in surgical skills across countries.

The insertion techniques also varied among respondents, with a significant proportion (41.1%) using manual instruments, while 36.7% reported using both manual and rotary instruments. The choice of instruments may reflect personal preferences or training, as well as the perceived control offered by manual methods during the delicate process of MI insertion. Additionally, manual instruments such as hand screwdrivers are typically more affordable, easier to maintain, and provide a more economical solution for practitioners in smaller or cost-sensitive practices. However, the landscape of orthodontic practices in Romania is evolving, with a growing trend toward large interdisciplinary clinics. Considering this, along with the increasing availability of rotary systems, which offer precision and efficiency, a shift toward a more widespread use of rotary instruments may be expected in the future [26].

The overall reported success rate of MI in this study was high, with 57.8% of respondents indicating success rates between 76% and 100%. This finding is consistent with previous studies, where MIs have demonstrated high success rates in providing stable anchorage for orthodontic treatment [27,28]. Despite this, complications were not uncommon, with MI mobility being the most frequently reported issue (92.2%), followed by soft tissue damage (57.8%) and pain or discomfort post-insertion (54.4%). These complications are well documented in the literature and often arise due to factors such as poor patient hygiene, improper insertion technique, or unfavorable anatomical conditions [29].

The high incidence of MI mobility may warrant further investigation into the factors influencing MI stability, particularly the role of insertion protocols and implant design. [30,31]. Moreover, the fact that soft tissue damage and post-insertion pain were common complications suggests that patient management during the post-operative period is crucial in minimizing discomfort and ensuring the success of the treatment. Future research could explore strategies to reduce these complications, including optimized insertion techniques and patient education on post-insertion care [14,32].

This study fills an important gap in the literature concerning the use of MIs among Romanian orthodontists, providing insights that could inform clinical practice and guide future training initiatives. The high prevalence of MI usage, combined with the diversity of systems and techniques employed, highlights the need for standardized protocols and continued professional development in this area. Given the increasing demand for complex orthodontic treatments, particularly in cases requiring skeletal anchorage, ensuring that practitioners have access to up-to-date training on MI insertion and management is essential.

In this study, only the items from Sections One, Two, and Four of the questionnaire were analyzed due to the large volume of data collected. The results from Section Three, which specifically addresses the dimensions of mini-implants used by orthodontists in various anatomical regions, will be examined in a separate article. This approach aims to provide a more in-depth analysis, allowing for a thorough exploration of how the dimensions of mini-implants influence treatment outcomes and how these parameters are tailored to meet the specific needs of individual patients.

Our study underscores the importance of integrating orthodontic MIs into routine clinical practice given their high success rates and the ability to manage complex cases effectively. Enhancing practitioner training on MI techniques could reduce complications, thus improving patient outcomes. This calls for a systematic inclusion of MI application training in dental education curricula and continuous professional development programs. Further research is needed to explore the long-term outcomes of MI usage and the factors influencing its success. Comparative studies across different demographic and economic settings could provide deeper insights into optimizing MI usage for diverse patient populations. Additionally, innovative studies focusing on the development of new MI materials and technologies could help address the current challenges noted in MI application, potentially leading to advancements in orthodontic treatment methodologies.

While this study provides valuable insights, some limitations must be acknowledged. First, this survey captures only a snapshot of MI usage at a specific point in time without accounting for changes in practice patterns over time. Longitudinal studies could offer a more dynamic understanding of how MI usage evolves among Romanian orthodontists. Additionally, this study relies on self-reported data, which may introduce bias, particularly in the assessment of success rates and complications. Future research could benefit from the inclusion of objective clinical data to validate these findings. Moreover, while this study focuses on Romanian practitioners, the results may have broader implications for other regions with similar orthodontic practices. Comparative studies across different countries could provide a more global perspective on MI usage and help identify best practices that could be implemented universally.

## 5. Conclusions

In conclusion, this study highlights the widespread use of MIs among Romanian orthodontists, with most practitioners using them to improve anchorage and manage complex cases. The study found a strong correlation between MI usage and the operator’s experience, with more experienced orthodontists utilizing MIs more frequently. While the overall success rate is high, common complications such as implant mobility and soft tissue damage suggest a need for better insertion protocols and post-operative care. These insights underscore the importance of continued professional development and further research to optimize MI usage in clinical practice.

## Figures and Tables

**Table 1:** Items and responses from Section Two.

Number of Item	Item	Options
5	Do you use orthodontic MI in your practice?	Yes
		No
6	How long have you been using MI in your practice?	<1 year
		1–3 years
		3–6 years
		6–10 years
		>10 years
		I don’t use MI
7	What orthodontic MI system do you use?	Dual Top (Jeil Medical)
		Benefit (PSM)
		OrthAnchor (OSSTEM)
		OrthoEasy (Forestadent)
		Leone (Leone)
		Tomas (Dentaurum)
		Vector TAS (ORMCO)
		Fatscrew (Air Orthodontics)
		Infinity (IOS Ortho)
		Aarhus (AO)
		Other (free question)
		I don’t use MI
8	Who applies the MI in your practice?	The orthodontist
		The oromaxillofacial surgeon
		The dentoalveolar surgeon
		The general dentist
		Other (open question)
		I don’t use MI
9	How do you evaluate the frequency of MI use in your practice?	Very frequently (9–10)
		Frequently (7–8)
		Occasionally (5–6)
		Rarely (3–4)
		Very rarely (1–2)
		I don’t use MI (0)
10	Which type of instruments do you use most frequently for MI insertion?	Manual instruments
		Rotary instruments
		Both types of instruments
		I don’t use MI
11	What clinical/paraclinical methods do you use to evaluate the site of MI insertion?	Only clinical examination
		Clinical examination and 2D
		Clinical examination and 3D
		Other methods (open question)
		I don’t use MI
12	What is the most frequently used area for the insertion of orthodontic MI in your practice?	Interradicular
		Palatal
		Midpalatal
		Infrazygomatic
		Retromolar
		Others (open question)
		I don’t use MI

**Table 2:** Items and responses from Section Four.

Number of Item	Item	Options
20	How satisfied are you with the following aspects of orthodontic MI? a. Planning and insertion of MI b. MI stability c. Efficiency in tooth movement d. Management of complications e. Cost of MI	Very satisfied (9–10)
		Satisfied (7–8)
		Indifferent (5–6)
		Dissatisfied (3–4)
		Very dissatisfied (1–2)
21	How do you evaluate the success rate of MI use in your practice?	0–25%
		26–50%
		51–75%
		76–100%
22	Why did you choose to use orthodontic mini-implants?	Better anchorage stability
		Ability to treat complex cases
		Increased treatment efficiency
		Reduction of patient discomfort
		Other reasons (open question)
23	How do you evaluate the overall risk of complications associated with orthodontic MI?	Very high (9–10)
		High (7–8)
		Medium (5–6)
		Low (3–4)
		Very low (1–2)
24	What complications/accidents have you encountered following the use of mini-implants?	MI mobility
		Soft tissue damage
		Hard tissue damage
		Pain and discomfort after insertion
		MI fracture
		Not encountered
		Others (open question)

**Table 3:** Distribution according to responses for items 1 to 4.

Variable	No.	Percentage
**Gender**		
Female	74	70.5%
Male	31	29.5%
**Specialization**		
Orthodontics	93	88.6%
Dento-alveolar surgery	5	4.8%
General dentistry	1	1.0%
Oral and maxillofacial surgery	0	0.0%
Other specialization	0	0.0%
Resident doctor	6	5.7%
**Experience**		
Less than 5 years	34	32.4%
5–10 years	19	18.1%
11–15 years	27	25.7%
16–20 years	12	11.4%
Over 20 years	13	12.4%
**City**		
Oradea	25	23.8%
Cluj-Napoca	19	18.1%
Timișoara	9	8.6%
Iași	5	4.8%
Târgu Mureș	5	4.8%
București	16	15.2%
Mediaș	1	1.0%
Ploiești	1	1.0%
Arad	2	1.9%
Brăila	2	1.9%
Craiova	3	2.9%
Buzău	3	2.9%
Bistrița	2	1.9%
Baia Mare	1	1.0%
Turda	4	3.8%
Pitești	4	3.8%
Sibiu	2	1.9%
Satu Mare	1	1.0%

**Table 4:** Distribution according to responses for items 5 to 12.

Answer	No.	Percentage
**Item 5: Do you use orthodontic MI in your practice?**		
No	15	14.3%
Yes	90	85.7%
**Item 6: How long have you been using MI in your practice?**		
<1 year	12	13.3%
1–3 years	24	26.7%
3–6 years	15	16.7%
6–10 years	16	17.8%
>10 years	23	25.6%
**Item 7: What orthodontic MI system do you use**		
Dual Top (Jeil Medical)	54	60%
Benefit (PSM)	39	43.3%
OrthAnchor (OSSTEM)	13	14.4%
OrthoEasy (Forestadent)	21	23.3%
Leone (Leone)	9	10.0%
Tomas (Dentaurum)	3	3.3%
Vector TAS (ORMCO)	4	4.4%
Fatscrew (Air Orthodontics)	5	5.6%
Infinity (IOS Ortho)	1	1.1%
Aarhus (AO)	0	0.0%
Other	8	8.9%
**Item 8: Who applies the MI in your practice?**		
The orthodontist	71	78.9%
The oromaxillofacial surgeon	13	14.4%
The dentoalveolar surgeon	36	40%
The general dentist	4	4.4%
Other	0	0.0%
**Item 9: How do you evaluate the frequency of MI use in your practice?**		
Very frequently	16	17.8%
Frequently	40	44.4%
Occasionally	24	26.7%
Rarely	9	10.0%
Very rarely	1	1.1%
**Item 10: Which type of instruments do you use most frequently for MI insertion?**		
Manual instruments	37	41.1%
Rotary instruments	20	22.2%
Both types of instruments	33	36.7%
**Item 11: What clinical/paraclinical methods do you use to evaluate the site of MI insertion?**		
Only clinical examination	69	76.7%
Clinical examination and 2D	65	72.2%
Clinical examination and 3D	63	70%
Other methods	3	3.3%
**Item 12: What is the most frequently used area for the insertion of orthodontic MI in your practice?**		
Interradicular	54	60.0%
Palatal	14	15.6%
Midpalatal	3	3.3%
Infrazygomatic	9	10.0%
Retromolar	6	6.7%
Others	4	4.4%

**Table 5:** Distribution according to system usage and MI placement.

MI Placement/System (Absent/Present)	Dual Top	Benefit (PSM)
Interradicular	28 (77.8%)/26 (48.1%)	34 (66.7%)/20 (51.3%)
Midpalatal	1 (2.8%)/2 (3.7%)	1 (2%)/2 (5.1%)
Retromolar	4 (11.1%)/2 (3.7%)	1 (2%)/5 (12.8%)
Infrazygomatic	2 (5.6%)/7 (13%)	7 (13.7%)/2 (5.1%)
Palatal	1 (2.8%)/13 (24.1%)	4 (7.8%)/10 (25.6%)
Others	0 (0%)/4 (7.4%)	4 (7.8%)/0 (0%)
*p* *	0.004	0.008
**MI Placement/System (Absent/Present)**	**Fatscrew—Air**	**Vector TAS**
Interradicular	53 (62.4%)/1 (20%)	54 (62.8%)/0 (0%)
Midpalatal	3 (3.5%)/0 (0%)	3 (3.5%)/0 (0%)
Retromolar	2 (2.4%)/4 (80%)	6 (7%)/0 (0%)
Infrazygomatic	9 (10.6%)/0 (0%)	7 (8.1%)/2 (50%)
Palatal	14 (16.5%)/0 (0%)	12 (14%)/2 (50%)
Others	4 (4.7%)/0 (0%)	4 (4.7%)/0 (0%)
*p* *	<0.001	0.028

* Fisher’s Exact Test. Results are presented as merged contingency tables; number of cases and percentages of MI placement zones are written as column values (reported to total number of cases with absence or presence of specific system usage).

**Table 6:** Distribution according to frequency and MI usage experience.

MI Experience/Usage	<1 Years	1–3 Years	3–6 Years	6–10 Years	>10 Years	*p* *
Very rarely	0 (0%)	1 (4.2%)	0 (0%)	0 (0%)	0 (0%)	<0.001
Rarely	1 (8.3%)	2 (8.3%)	4 (26.7%)	2 (12.5%)	0 (0%)	
Occasionally	6 (50%)	9 (37.5%)	4 (26.7%)	5 (31.3%)	0 (0%)	
Frequently	5 (41.7%)	8 (33.3%)	6 (40%)	7 (43.8%)	14 (60.9%)	
Very frequently	0 (0%)	4 (16.7%)	1 (6.7%)	2 (12.5%)	9 (39.1%)	

* Fisher’s Exact Test. Frequency of MI usage categories are written as row headers, while experience with MI usage categories are written as column headers. Data are presented as number of cases with percentages, written as column values (reported to total number of cases with specific MI frequency usage).

**Table 7:** Distribution according to responses provided for items 20 to 24.

Answer	No.	Percentage
**Item 20: How satisfied are you with the following aspects of orthodontic MI?**		
(**a**) Planning and insertion of MI		
Very satisfied	38	42.2%
Satisfied	49	54.4%
Indifferent	2	2.2%
Dissatisfied	1	1.1%
Very dissatisfied	0	0.0%
(**b**) MI stability		
Very satisfied	12	13.3%
Satisfied	64	71.1%
Indifferent	8	8.9%
Dissatisfied	6	6.7%
Very dissatisfied	0	0.0%
(**c**) Efficiency in tooth movement		
Very satisfied	54	60%
Satisfied	34	37.8%
Indifferent	2	2.2%
Dissatisfied	0	0.0%
Very dissatisfied	0	0.0%
(**d**) Management of complications		
Very satisfied	20	22.2%
Satisfied	62	68.9%
Indifferent	4	4.4%
Dissatisfied	4	4.4%
Very dissatisfied	0	0.0%
(**e**) Cost of MI		
Very satisfied	17	18.9%
Satisfied	54	60%
Indifferent	10	11.1%
Dissatisfied	7	7.8%
Very dissatisfied	2	2.2%
**Item 21: How do you evaluate the success rate of MI use in your practice?**		
0–25%	0	0.0%
26–50%	6	6.7%
51–75%	32	35.6%
76–100%	52	57.8%
**Item 22: Why did you choose to use orthodontic mini-implants?**		
Better anchorage stability	70	77.8%
Ability to treat complex cases	68	75.6%
Increased treatment efficiency	53	58.9%
Reduction of patient discomfort	16	17.8%
Other reasons	0	0.0%
**Item 23: How do you evaluate the overall risk of complications associated with orthodontic MI?**		
Very high	0	0.0%
High	1	1.1%
Medium	27	30.0%
Low	53	58.9%
Very low	9	10.0%
**Item 24: What complications/accidents have you encountered following the use of mini-implants?**		
MI mobility	83	92.2%
Soft tissue damage	52	57.8%
Hard tissue damage	10	11.1%
Pain and discomfort after insertion	49	54.4%
MI fracture	19	21.1%
Not encountered	0	0.0%
Others	0	0.0%

**Table 8:** Distribution according to MI placement and overall risk of complications.

Risk/Placement	Interradicular	Midpalatal	Retromolar	Infrazygomatic	Palatal	Others	*p* *
Very low	6 (11.1%)	0 (0%)	2 (33.3%)	1 (11.1%)	0 (0%0	0 (0%)	<0.001
Low	32 (59.3%)	2 (66.7%)	4 (66.7%)	0 (0%)	11 (78.6%)	4 (100%)	
Medium	16 (29.6%)	0 (0%)	0 (0%)	8 (88.9%)	3 (21.4%)	0 (0%)	
High	0 (0%)	1 (33.3%)	0 (0%)	0 (0%)	0 (0%)	0 (0%)	

* Fisher’s Exact Test. Data are presented as number of cases with percentages, written as column values (reported to total number of cases with specific MI placement zones).

**Table 9:** Distribution according to system usage and MI placement.

MI Placement/Complication (Absent/Present)	Fracture	Hard Tissue Damage
Interradicular	45 (63.4%)/9 (47.4%)	51 (63.7%)/3 (30%)
Midpalatal	3 (4.2%)/0 (0%)	2 (2.5%)/1 (10%)
Retromolar	1 (1.4%)/5 (26.3%)	6 (7.5%)/0 (0%)
Infrazygomatic	7 (9.9%)/2 (10.5%)	9 (11.3%)/0 (0%)
Palatal	11 (15.5%)/3 (15.8%)	12 (15%)/2 (20%)
Others	4 (5.6%)/0 (0%)	0 (0%)/4 (40%)
*p* *	0.016	<0.001

* Fisher’s Exact Test. Results are presented as merged contingency tables; number of cases and percentages of MI placement zones are written as column values (reported to total number of cases with absence or presence of specific complication).

**Table 10:** Distribution according to MI placement and overall success rate.

Success/Placement	Interradicular	Midpalatal	Retromolar	Infrazygomatic	Palatal	Others	*p* *
26–50%	4 (7.4%)	0 (0%)	0 (0%)	0 (0%)	2 (14.3%)	0 (0%)	0.038
51–75%	17 (31.5%)	3 (100%)	0 (0%)	6 (66.7%)	6 (42.9%)	0 (0%)	
76–100%	33 (61.1%)	0 (0%)	6 (100%)	3 (33.3%)	6 (42.9%)	4 (100%)	

* Fisher’s Exact Test. Data are presented as number of cases with percentages, written as column values (reported to total number of cases with specific MI placement zones).

## Data Availability

The data presented in this study are available upon request from the corresponding authors. The data are not publicly available due to privacy reasons.
